# Low-Density Lipoprotein Subfraction Phenotype Is Associated with Epicardial Adipose Tissue Volume in Type 2 Diabetes

**DOI:** 10.3390/jcm14030862

**Published:** 2025-01-28

**Authors:** José Rives, Pedro Gil-Millan, David Viladés, Álvaro García-Osuna, Idoia Genua, Inka Miñambres, Margarida Grau-Agramunt, Ignasi Gich, Nuria Puig, Sonia Benitez, Josep Julve, Antonio Pérez, José Luis Sánchez-Quesada

**Affiliations:** 1 Cardiovascular Biochemistry, Institut de Recerca Sant Pau (IR-Sant Pau), 08041 Barcelona, Spain; jrives@santpau.cat (J.R.); agarciao@santpau.cat (Á.G.-O.); mgrauag@santpau.cat (M.G.-A.); npuigg@santpau.cat (N.P.); sbenitez@santpau.cat (S.B.); 2 Department of Biochemistry and Molecular Biology, Universitat Autònoma de Barcelona, 08193 Barcelona, Spain; 3 Endocrinology Department, Hospital de la Santa Creu i Sant Pau, 08041 Barcelona, Spain; pedrogilmillan18@gmail.com (P.G.-M.); igenua@santpau.cat (I.G.); iminambres@santpau.cat (I.M.); jjulve@santpau.cat (J.J.); 4 Department of Medicine, Universitat Autònoma de Barcelona, 08193 Barcelona, Spain; 5 Cardiology Department, Hospital de la Santa Creu i Sant Pau, 08041 Barcelona, Spain; dvilades@santpau.cat; 6 CIBER of Cardiovascular Diseases (CIBERCV), Madrid, Spain; 7 CIBER of Diabetes and Metabolic Diseases (CIBERDEM), Madrid, Spain; 8 Epidemiology Department, Hospital de la Santa Creu i Sant Pau, 08041 Barcelona, Spain; igich@santpau.cat; 9 CIBER Epidemiología y Salud Pública (CIBERESP), Madrid, Spain

**Keywords:** type 2 diabetes, coronary artery disease, epicardial adipose tissue, LDL size, hepatic function

## Abstract

**Background**: Increased epicardial adipose tissue (EAT) volume is a common feature in type 2 diabetes (T2DM) which is directly associated with heart failure and advanced atherosclerosis. We aimed to evaluate lipoprotein-related biomarkers of EAT volume in T2DM patients before and after glycemic control. **Methods**: This study included 36 T2DM patients before and after optimization of glycemic control and on 14 healthy controls (HCs). EAT volume was measured using computed tomography imaging indexed to the body surface area (iEAT). Biochemical and lipid profiles were determined using commercial methods. Lipoproteins were isolated by ultracentrifugation, and variables of lipoprotein function were assessed. Multivariable regression analysis was used to find variables independently associated with iEAT. **Results**: iEAT was higher in T2DM than in controls and decreased with glycemic optimization. HDLs from T2DM had less apoA-I and cholesterol and more apoC-III and triglycerides. LDLs from T2DM had more triglycerides and apoB and smaller sizes than those from HCs. Significant correlations were found between iEAT and age, BMI, HbA1c, GGT, VLDLc, triglycerides, LDL size, apoA-I in HDL, and apoC-III in HDL. In the multivariable regression analysis, age, LDL size, and GGT associations remained statistically significant, and predicted 50% of the variability in EAT volume. ROC analysis using these variables showed an AUC of 0.835. **Conclusions**: Qualitative characteristics of lipoproteins were altered in T2DM. Multivariable analysis showed that LDL size and GGT plasma levels were independently associated with iEAT volume, suggesting that these variables might be useful biomarkers for stratifying T2DM patients with increased EAT volume.

## 1. Introduction

Cardiovascular disease, either expressed as coronary artery disease (CAD) or heart failure (HF), is the leading cause of mortality in individuals with type 2 diabetes mellitus (T2DM) [1]. Excessive adiposity is involved in the development of T2DM and atherosclerotic vascular diseases. In this situation, there is an increased release of nonesterified fatty acids (NEFAs) in the bloodstream due to alterations in the activities of lipoprotein lipase (LPL) and hormone-sensitive lipase (HSL) in visceral adipose tissue, and these NEFAs are taken up by the liver. These alterations ultimately lead to insulin resistance, diabetes, and atherogenic dyslipidemia. This results in increased hepatic synthesis of apolipoprotein B (apoB) and very low-density lipoprotein (VLDL), which is the beginning of the entity known as diabetic or atherogenic dyslipidemia [2,3]. Besides the characteristic features of atherogenic dyslipidemia (hypertriglyceridemia, decreased levels of high-density lipoprotein [HDL] cholesterol, and increased levels of small dense low-density lipoprotein [LDL] particles), there are also changes in the composition of lipoproteins that contribute to altered functionality and greater atherogenic potential [3,4,5].

Epicardial adipose tissue (EAT) is an ectopic fat deposit located between the visceral pericardium and myocardium surrounding the coronary arteries and the cardiac nervous system. EAT is more metabolically active than subcutaneous or pericardial fat, and its smaller adipocytes secrete a wide range of adipokines, bioactive lipids, microRNAs, and other molecules [6]. In a healthy state, EAT has a protective anti-inflammatory function and acts as a buffer for NEFAs, thus protecting surrounding tissues from the deleterious effects of excessive NEFA concentration. EAT volume has been shown to increase in T2DM and in other metabolic disorders, such as obesity, metabolic syndrome, and hypertension, and is associated with an elevated risk of cardiovascular disease [7,8]. In addition, EAT is not only a biomarker of cardiovascular risk but also an active participant in the development of CAD. A large EAT volume is accompanied by dysfunctional features, such as increased adipocyte size, greater macrophage infiltration, a higher proportion of M1 proinflammatory macrophages, imbalance in the secretion of proinflammatory mediators, and elevated levels of reactive oxygen species (ROS) and NEFAs, particularly in patients with diabetes [9]. Specifically, pericoronary fat, a subtype of EAT that surrounds coronary arteries, has been related with increased major adverse coronary events and altered inflammatory balance in prediabetic patients [10]. In conjunction, the accumulation of EAT promotes endothelial dysfunction and the development of CAD [11,12,13,14,15].

EAT volume can be assessed using imaging techniques such as echocardiography, cardiac multidetector computed tomography (MDCT), and magnetic resonance imaging (MRI) [16,17,18]. However, these techniques are not easily applicable due to the need for specialized facilities and trained personnel and because MDCT and MRI specifically are very expensive. Unfortunately, there are no reliable plasma biomarkers to determine the accumulation of EAT or that could complement image studies.

The relationship between lipoprotein metabolism dysfunctions and the increase in the volume of EAT has been poorly studied until now [19], and no clear links have been established between the two phenomena, which coexist in pathologies derived from metabolic dysfunctions, such as diabetes. In a simplistic view, it can be considered that diabetic dyslipidemia contributes to the development of early coronary atherosclerosis in a systemic manner, while EAT acts locally as a transducer of a proinflammatory environment. However, it is unclear how lipoprotein metabolism and EAT interact with each other or whether their influence on CAD development is related or independent. Our research aims to investigate the relationship between lipoprotein metabolism and EAT accumulation to gain a better understanding of EAT’s role in the development of cardiovascular disease in T2DM patients. We expect to find parameters of the lipoprotein metabolism associated with EAT accumulation that could help to better stratify T2DM patients with higher cardiovascular risk and to reduce as much as possible the need for expensive and complex imaging techniques.

## 2. Materials and Methods

### 2.1. Study Design, Setting and Subjects’ Characteristics

Patients first diagnosed with T2DM (n = 36) were recruited from 2017 to 2020 and followed up by the Endocrinology and Nutrition Department at the Hospital de la Santa Creu i Sant Pau. Diabetes was defined according to the criteria of the American Diabetes Association [20]. MDCT, MRI, and blood extraction for laboratory routine and lipoprotein analyses were performed in 36 patients at diagnosis and after 12 months of glycemic control optimization. Clinical data were recorded by anamnesis and a review of the patient’s medical history before and after glycemic control optimization. Anthropometric measurements, such as body mass index (BMI), weight, height, and waist circumference (WC), were recorded. Hypertension, dyslipidemia, and smoking status were also noted (Table 1). Hypertension was defined according to the criteria established by the European Society of Hypertension/European Society of Cardiology [21]. Dyslipidemia was defined as the presence of any of the following: triglyceride concentrations of 1.7 mmol/L or greater, HDLc concentrations of less than 1 mmol/L in men or less than 1.3 mmol/L in women, or LDLc concentrations greater than 4.14 mmol/L. None of the patients had prior documented diagnosis of cardiovascular or renal disease. Patients undergoing pathologies or lipid-lowering/anti-inflammatory treatments that could affect lipoprotein metabolism were excluded. Glycemic control optimization was achieved by multifactorial intervention, including weight control, diet, physical activity, and pharmacotherapy, according to clinical practice guidelines. In cases of severe hyperglycemia, the initial therapy included metformin, dipeptidyl peptidase inhibitors (DPP4i), glucagon-like peptide-1 agonists (GLP1-rA), and basal insulin in 90% of the patients. Basal insulin was suspended after 2 weeks, and noninsulin pharmaceutical treatment was modified based on the individualized characteristics of the patients. Treatment with Sodium-glucose cotransporter-2 inhibitor (SGLT2-I, Empaglifozin) was initiated at 2 weeks after an improvement of the severe hyperglycemia, based on our hospital’s protocol. The improvement of glycemic control was achieved in a period of 3–6 months.

Fourteen healthy volunteers matched for age, gender, and smoking habits were selected as the control group. They underwent MDCT and MRI, and their blood was drawn for analysis. Individuals who had a personal or family history of premature coronary artery disease; had major cardiovascular risk factors, such as smoking, dyslipidemia, or diabetes; had a recent history of infectious or inflammatory disease within the last three months; or were taking anti-inflammatory drugs were not included. The study was approved by the Ethics Committee of the Hospital de Sant Pau (IIBSP-REL-2017-27). Written informed consent was obtained from all participants. This study was performed in full compliance with the Declaration of Helsinki. This study followed STROBE guidelines.

### 2.2. Image Analysis

Cardiac MRI was performed using a 3 Tesla scanner (Siemens Magnetom Vida, Siemens Healthineers, Erlangen, Germany). After obtaining the usual scout planes, steady-state free-precession cine-MR images were acquired in individual long-axis planes and in multiple 8-mm-thick short-axis slices with a 2-mm gap from the atrioventricular groove to the apex of the left and right ventricle. T1 and T2 mapping sequences were also obtained in three short-axis planes (basal, mid, and apical) using the same orientation as the cine-MRI images. A 3D inversion recovery segmented gradient echo sequence was acquired 10 min after the administration of gadobutrol contrast (0.2 mmol/kg; Gadovist 1.0, Bayer Schering Pharma, Berlin, Germany) to assess the presence of myocardial delayed enhancement (DE). Inversion times were adjusted to nullify the signal from the normal myocardium (200–300 ms). This sequence was acquired during a breath hold of approximately 20 s in multiple short-axis planes using the same orientation as the cine-MR images. The main duration of the CMR study was 30 min, and the analysis of the imaging data was performed by one specialized cardiologist (DVM) blinded to the clinical data. Cine loops and contrast-enhanced images were analyzed using dedicated software (QMass MR 7.1, MEDIS, Leiden, The Netherlands). Left ventricular endocardial and epicardial borders were delineated semi-automatically on systolic and diastolic short-axis cine images. Left ventricular ejection fraction (LVEF) was measured in each patient. An LVEF of less than 50% was considered LV systolic dysfunction, according to acute and chronic heart failure guidelines [23].

MDCT and cardiac MRI studies were conducted on all subjects. MDCT was performed using a wide-coverage CT scanner (Aquilion One Vision 640 slices, Canon Medical Systems, Otawara, Japan). Coronary artery calcification was assessed from noncontrast CT scans according to the internationally agreed protocol [22], and the coronary artery calcium (CAC) burden was assessed using the Agatston score. Contrast cardiac CT scans were acquired prospectively in 75% of the RR interval with the following technical parameters: collimation 320 × 0.5 mm, rotation time 275 ms, and kilovoltage 100–120 kV. An intravenous contrast bolus (Xenetix 350 mg/mL, Guerbet, Villepinte, France) was administered at a rate of 1 mL/kg, followed by a bolus of physiological serum (40 mL), both at 6 mL/s. All MDCT studies were analyzed on an offline workstation by one specialized cardiologist (DVM) blinded to the study groups. EAT was calculated in the MDCT studies by drawing regions of interest on the different axial planes obtained after the upper and lower slice limits had been manually defined. Contiguous three-dimensional voxels from the limits of −150 to −30 Hounsfield units were defined as EAT voxels. The volume of adipose tissue in cubic centimeters (cm^3^) was obtained by adding the mapped areas using OsiriX MD (v1.2, FDA-cleared, Pixmeo, Geneva, Switzerland) software. Subsequently, EAT was reported in cm^3^ (EAT volume) and indexed to the body surface area (EAT index cm^3^/m^2^).

### 2.3. Biochemical Analysis

Blood was obtained using Vacutainer™ tubes (Becton Dickinson, Franklin Lakes, NJ, USA) without additives (serum) or EDTA-containing tubes (plasma). Serum and plasma were obtained by centrifugation for 15 min at 1500× *g* at room temperature. Glucose, total bilirubin, liver function (gamma glutamyl transferase [GGT], aspartate aminotransferase [AST], alanine transaminase [ALT], and alkaline phosphatase [ALP]), high sensitivity C-reactive protein (hsCRP), and lipid profile (cholesterol, triglycerides, VLDLc, LDLc, HDLc, and Lp(a)) were routinely analyzed in serum using an Alinity c autoanalyzer (Abbott Laboratories, Abbott Park, IL, USA). C-peptide, high-sensitive troponin T, and N-terminal pro B-type natriuretic peptide were quantified by electrochemiluminescence immunoassays in a Cobas e601 autoanalyzer (Roche Diagnostics, Basel, Switzerland). Glycated hemoglobin A1c (HbA_1c_) was measured from the total blood using ion-exchange high-performance liquid chromatography (Variant, Bio-Rad Laboratories, Hercules, CA, USA).

### 2.4. Lipoprotein Isolation and Composition

The following lipoproteins were isolated from plasma by ultracentrifugation using potassium bromide gradients: VLDL (density below 1.019 g/mL, including IDL), LDL (density range 1.019–1.063 g/mL), and HDL (density range 1.063–1.210 g/mL) [24]. Their lipid and apolipoprotein compositions were determined by measuring the content of cholesterol, triglycerides, apoB, apoA-I (Roche Diagnostics, Basel, Switzerland), phospholipids, free cholesterol (Wako Pure Chemical, Osaka, Japan), apoA-II, apoE, and apoC-III (Kamiya Biomedicals, Seattle, WA, USA) using a Cobas 6000/c501 autoanalyzer.

### 2.5. Lipoprotein Functional Assays

#### 2.5.1. Electronegative LDL

The proportion of electronegative LDL (LDL(-)) in the total LDL fraction was quantified by anion exchange step-wise chromatography using a MonoQ 5/50 GL column in an FPLC system (GE Healthcare, Chicago, IL, USA) at a flow of 1 mL/min, as previously described [25].

#### 2.5.2. LDL Susceptibility to Aggregation

The aggregation of LDL was induced by sphingomyelinase (SMase) lipolysis [26]. LDL was dialyzed against phosphate-buffered saline (PBS) at pH 7.4 using gel filtration chromatography in a PD10 column (Sephadex G-25, GE Healthcare). LDL (0.2 mmol/L cholesterol) was incubated at 37 °C for 2 h with SMase from *Bacillus cereus* sp. (Sigma Diagnostics, Livonia, MI, USA), with a final concentration of 25 mU/mL in a solution of 2 mM CaCl_2_ and 2 mM MgCl_2_. To separate aggregated LDL from nonaggregated LDL, size-exclusion chromatography was performed using a Superose 6 Increase 5/150 GL column (GE Healthcare) in an NGC system (Bio-Rad, Hercules, CA, USA) at a flow of 0.3 mL/min, as described in [27].

#### 2.5.3. LDL and HDL Susceptibility to Oxidation

Susceptibility to oxidation was assessed by dialyzing LDL and HDL against phosphate-buffered saline (PBS) at a pH of 7.4 using gel filtration chromatography in a PD10 column (Sephadex G-25, GE Healthcare). Oxidation was measured by continuous monitoring of the formation of conjugated dienes at 234 nm for 3 h using a Synergy HT spectrophotometer (BioTek-Agilent, Santa Clara, CA, USA) [28]. LDL and HDL, each at a cholesterol concentration of 0.2 protein/L, were incubated with 10 μM of CuSO_4_, and the lag phase time of the oxidation kinetics was determined. The antioxidative capacity of HDL in T2DM patients and HCs was evaluated by incubating HDL with a standard LDL (obtained from a pool of plasma with normal lipid levels and stored with 10% sucrose at −80 °C) and inducing oxidation by adding 10 μM of CuSO_4_. The results are expressed as the proportion of change in the lag phase time of standard LDL when compared to LDL alone, indicating increased or decreased HDL antioxidative activity [28].

#### 2.5.4. PAF-AH Activity

Platelet-activating factor acetylhydrolase (PAF-AH, also known as lipoprotein-associated phospholipase A2 (Lp-PLA_2_)) activity was assessed using 2-thio-PAF (Cayman Chemicals, Ann Arbor, MI, USA) as a substrate in accordance with the manufacturer’s instructions. The distribution of PAF-AH in the HDL fraction and plasma was determined by precipitating apoB-containing lipoproteins from the plasma using dextran sulfate, as described in [5].

#### 2.5.5. LDL Size and HDL Subfraction

LDL size and HDL subfraction proportions were analyzed from plasma using nondenaturing polyacrylamide gradient (2.5–16%) gel electrophoresis, as previously described [29].

### 2.6. Statistical Analysis

Continuous variables are expressed as mean ± SD (variables with parametric distribution) or median ± IQR (non-parametric variables). Categorical variables are expressed as percentages. The Kolmogorov–Smirnov test was used to assess data distribution normality. Comparisons between groups were performed using Student’s *t*-test, paired Student’s *t*-Test, Mann–Whitney U-test, or Wilcoxon signed-rank test, when appropriate. To analyze the correlation between iEAT and biochemical variables, Spearman’s rank correlation test was used. Bivariate and multivariable regression analysis were used to study variables independently associated with iEAT accumulation. Regression analysis was performed after transformation of the variables without normal distribution. Significant variables associated with iEAT in the bivariate regression analysis were included in the forward stepwise multivariable linear regression analysis. Receiver operating characteristic (ROC) analysis was used to determine the sensitivity and specificity of independently associated biomarkers to discriminate iEAT of 68.1 cm^3^/m^2^ or greater (95th percentile in the general population, according to Shmilovich et al. [17]). A *p*-value of less than 0.05 (two-sided) was considered statistically significant. The statistical software packages IBM-SPSS 29.0 for Windows (SPSS Inc.), GraphPad Prism Software 9.5 (GraphPad, Boston, MA, USA) and MedCalc^®^ Statistical Software 22.021 were used for statistical analyses.

## 3. Results

### 3.1. Clinical Characteristics and Biochemical Profiles of T2DM Patients and Healthy Controls

The patients’ clinical and anthropometric characteristics are shown in Table 1. Gender proportion and age were similar across the groups. Anthropometric measurements (BMI, weight, WC) were higher in the T2DM patients, showing a statistically significant decrease after 12 months of glycemic control optimization. Dyslipidemia and HTA were more frequent in the T2DM group (approximately one-third of the patients) and decreased after glycemic optimization. None of the patients with T2DM suffered major complications during the period of the study.

Table 2 provides a summary of the routine biochemical profiles. T2DM patients exhibited elevated HbA1c and blood glucose levels in comparison to healthy controls. After undergoing glycemic optimization, there was a significant improvement in these parameters. Hepatic enzyme levels were also altered in T2DM patients at baseline compared to healthy controls, but they decreased after good glycemic control. In addition, higher CRP levels were found in T2DM patients who showed a proinflammatory status, especially with poor glycemic control, which partly subsided after glycemic optimization.

### 3.2. Lipid Profile and Apolipoproteins in Plasma

No significant differences between the groups were observed in total cholesterol, LDLc, or Lp(a) levels. In T2DM patients, increased levels of triglycerides and VLDLc at baseline remained unchanged despite glycemic control. HDLc, apoA-I, and apoA-II levels were lower in T2DM patients at baseline compared to HCs and showed an increase after metabolic control had been achieved (Table 2). Plasma apoB was increased in PGC patients compared with healthy subjects but did not significantly change after glycemic optimization. No significant differences between the groups were found in apoC-III or apoE levels.

### 3.3. HF Biomarkers

**Figure 1:**
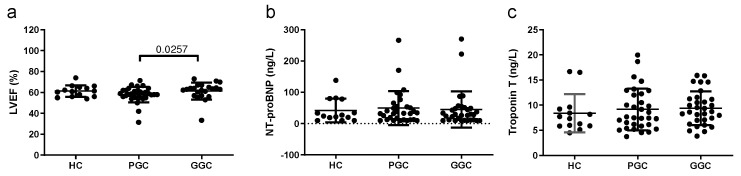
Scatter plots of heart failure markers. (**a**) LEVF, (**b**) NT-pro-BNP, and (**c**) hsTnT. LEVF was determined by MRI, and NT-proBNP and TnT were quantified in an autoanalyzer, as described in the Methods section. HC: healthy controls; PGC: T2DM patients with poor glycemic control; GGC: T2DM patients with good glycemic control. The horizontal bar indicates statistically significant differences. Data are expressed as mean ± SD.

LVEF data obtained by cardiac MRI were used to assess the presence of overt HF. Neither T2DM nor HC subjects had previous evidence of HF before or after metabolic control optimization, as shown in Figure 1. LVEF was not different between T2DM patients and HCs, although it showed a slight increase after the optimization of glycemic control. Both cardiac troponin T and NT-proBNP levels were in the non-pathological range (below 40 ng/L, and 450 ng/L, respectively) and did not show significant differences between T2DM patients and HCs, neither differed after metabolic control.

### 3.4. iEAT and CAC

**Figure 2:**
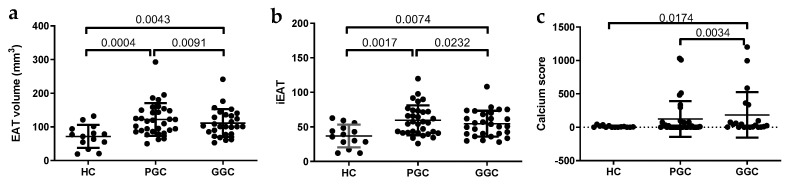
Scatter plots of EAT volume and CAC score. (**a**) EAT volume, (**b**) indexed EAT (iEAT), and (**c**) coronary calcium (CAC) score were obtained from MDCT analysis, as described in the Methods section. HC: healthy controls; PGC: T2DM patients with poor glycemic control; GGC: T2DM patients with good glycemic control. Horizontal bars indicate statistically significant differences between the groups. Data are expressed as mean ± SD.

EAT volume and EAT indexed to body surface area (iEAT) were larger in T2DM patients compared to HCs and decreased after 12 months of glycemic control (Figure 2). The CAC score was also higher in T2DM patients compared to HCs and showed a slight increase after glycemic control optimization (Figure 2).

### 3.5. Lipoprotein Composition

**Figure 3:**
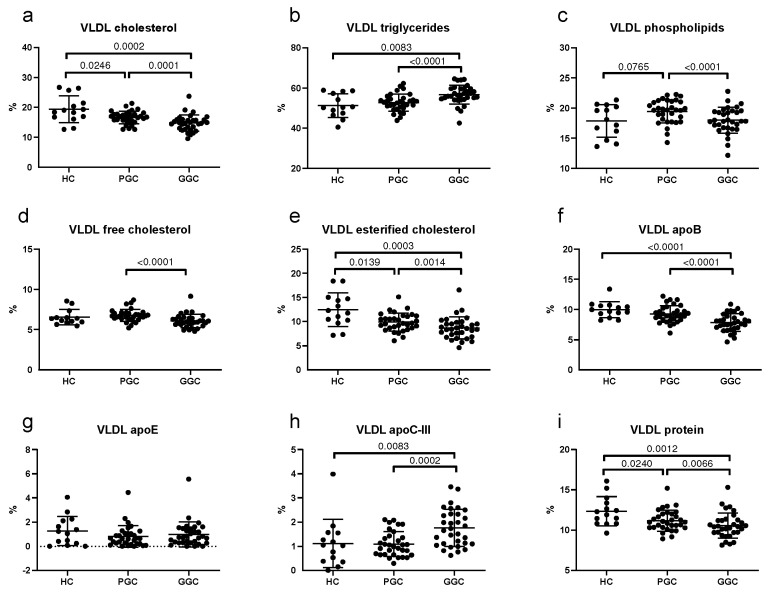
Scatter plots of VLDL composition. (**a**) total cholesterol, (**b**) triglycerides, (**c**) phospholipids, (**d**) free cholesterol, (**e**) esterified cholesterol, (**f**) apoB, (**g**) apoE, (**h**) apoC-III, (**i**) total protein. HC: healthy controls; PGC: T2DM patients with poor glycemic control; GGC: T2DM patients with good glycemic control. Horizontal bars indicate statistically significant differences between the groups. Composition was determined as described in the Methods section, and the results are expressed as the percentage of each component in the total mass of the lipoprotein. The horizontal bars indicate statistically significant differences. Data are mean ± SD.

**Figure 4:**
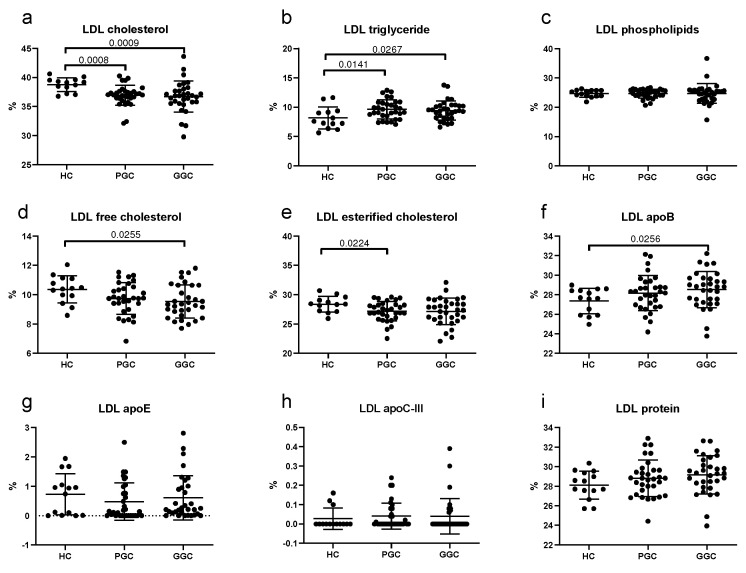
Scatter plots of the LDL composition. (**a**) total cholesterol, (**b**) triglycerides, (**c**) phospholipids, (**d**) free cholesterol, (**e**) esterified cholesterol, (**f**) apoB, (**g**) apoE, (**h**) apoC-III, (**i**) total protein. HC: healthy controls; PGC: T2DM patients with poor glycemic control; GGC: T2DM patients with good glycemic control. Horizontal lines indicate statistically significant differences between the groups. Composition was determined as described in the Methods section, and the results are expressed as the percentage of each component in the total mass of the lipoprotein. The horizontal bars indicate statistically significant differences. Data are mean ± SD.

**Figure 5:**
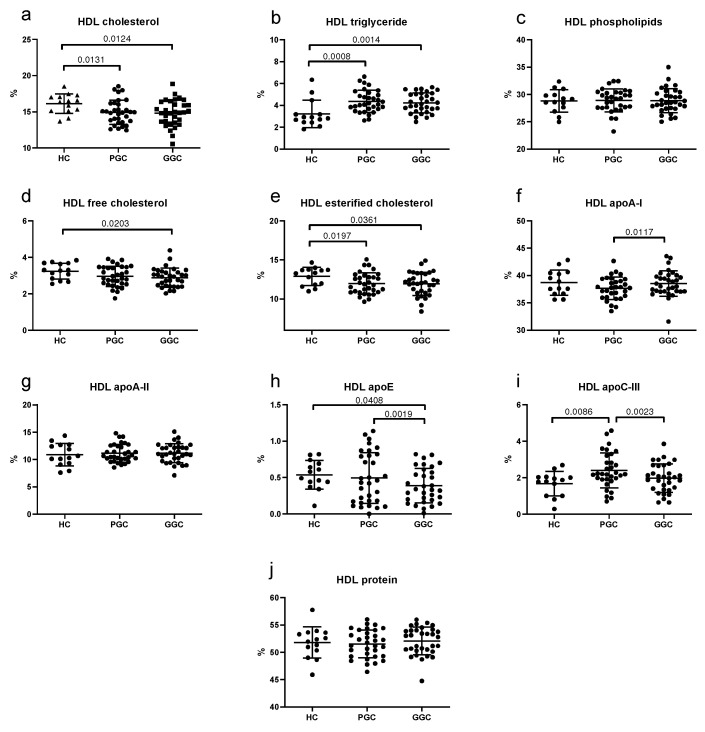
Scatter plots of HDL composition. (**a**) total cholesterol, (**b**) triglycerides, (**c**) phospholipids, (**d**) free cholesterol, (**e**) esterified cholesterol, (**f**) apoA-I, (**g**) apoA-II, (**h**) apoE, (**i**) apoC-III, (**j**) total protein. HC: healthy controls; PGC: T2DM patients with poor glycemic control; GGC: T2DM patients with good glycemic control. Horizontal lines indicate statistically significant differences between the groups. Composition was determined as described in the Methods section, and the results are expressed as the percentage of each component in the total mass of the lipoprotein. The horizontal bars indicate statistically significant differences. Data are mean ± SD.

Alterations in the composition of VLDL, LDL, and HDL are shown in Figure 3, Figure 4 and Figure 5, respectively. Regarding VLDL, these lipoproteins in T2DM patients with poor glycemic control were relatively enriched in triglycerides and had less cholesterol, apoB, and protein content compared with healthy controls, which indicates that VLDL particles were larger in T2DM patients at baseline (Figure 3). Such alterations remained unchanged after metabolic control.

LDLs in T2DM patients at baseline showed a lower content of total cholesterol but higher levels of triglycerides and apoB compared with control subjects (Figure 4), suggesting smaller and denser LDL particles. This difference in particle size was confirmed by gradient gel electrophoresis (see the next section).

HDLs from T2DM patients at baseline exhibited a higher proportion of triglycerides and apoC-III, but lower levels of cholesterol and apoA-I compared to HCs (Figure 5). The improvement in glycemic control normalized the content of apoA-I and apoC-III in HDLs from T2DM patients.

### 3.6. Lipoprotein Functional Properties

**Figure 6:**
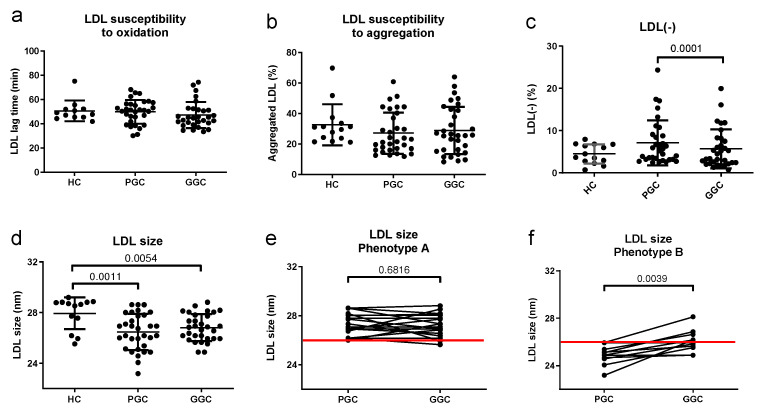
Functional LDL properties. LDL susceptibility to in vitro-induced oxidation (**a**) and aggregation (**b**), proportion of LDL(-) (**c**), and LDL size (**d**) were assessed as described in the Methods section. Patients were classified as LDL subfraction phenotype A or B when the LDL particle size was higher (**e**) or lower (**f**) than 26.0 nm. The red line in (**e**,**f**) indicates a threshold of 26.0 nm. HC: healthy controls; PGC: T2DM patients with poor glycemic control; GGC: T2DM patients with good glycemic control. Horizontal bars indicate statistically significant differences between the groups. Data are expressed as mean ± SD.

From a functional point of view, neither LDL susceptibility to oxidation nor susceptibility to aggregation was altered in the T2DM patients compared with the healthy subjects (Figure 6a,b). In contrast, LDL from T2DM patients with poor glycemic control was smaller and had an increased proportion of LDL(-) than LDL from healthy controls (Figure 6c). The LDL(-) proportion in T2DM patients decreased after glycemic optimization, but the LDL size was not modified after treatment when all subjects were analyzed (Figure 6d). However, when patients were classified according to LDL subfraction phenotype A or B (LDL particle size higher or lower than 26.0 nm), those with small LDL (phenotype B, <26.0 nm) at baseline increased particle size after glycemic control (Figure 6e,f).

**Figure 7:**
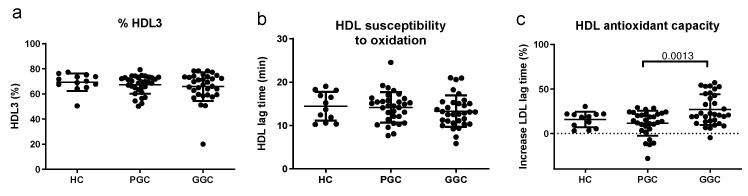
Functional HDL properties. HDL3 proportion (**a**), susceptibility to in vitro-induced oxidation (**b**), and capacity to prevent LDL oxidation (**c**) were assessed as described in the Methods section. HC: healthy controls; PGC: T2DM patients with poor glycemic control; GGC: T2DM patients with good glycemic control. The horizontal bars indicate statistically significant differences between the groups. Data are expressed as mean ± SD.

No difference in HDL size (expressed as the proportion of HDL3, the subfraction with the lowest size) or HDL susceptibility to oxidation was observed between the groups (Figure 7). HDLs in T2DM patients at baseline had a lower capacity to prevent LDL oxidation than after the optimization of metabolic control.

**Figure 8:**
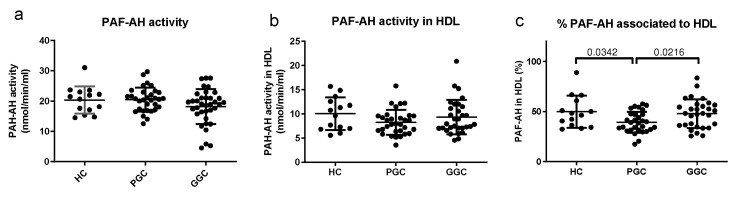
Total PAF-AH activity in plasma and content in HDL. Total PAF-AH activity in plasma (**a**), PAF-AH activity associated with HDL (**b**), and proportion of PAF-AH activity in HDL (**c**). PAF-AH activity was assessed as described in the Methods section. HC: healthy controls; PGC: T2DM patients with poor glycemic control; GGC: T2DM patients with good glycemic control. The horizontal bars indicate statistically significant differences between the groups. Data are expressed as mean ± SD.

Regarding PAF-AH activity and distribution, no difference in total activity was observed between the groups (Figure 8). However, the proportion of PAF-AH transported by HDL was decreased in T2DM patients at baseline and increased after glycemic optimization.

### 3.7. Correlations with iEAT

Supplemental File S1 shows the correlations between all parameters, analyzed using Spearman’s rank correlation coefficient test. All subjects were included in this analysis. Significant correlations are marked in yellow. For easier interpretation, Table 3 summarizes the correlations found between the iEAT and HDL-related variables that showed statistically significant differences between DM and HC groups, and Table 4 summarizes those between the iEAT and LDL-related variables. The rest of the correlations are shown in File S1. Regarding anthropometric measurements, basic biochemistry and lipid profile, age (*p* < 0.001, r = 0.442), BMI (*p* = 0.003, r = 0.343), HBA_1c_ (*p* = 0.006, r = 0.326), GGT (*p* < 0.001, r = 0.456), VLDLc (*p* = 0.032, r = 0.255) and total triglycerides (*p* = 0.0480, r = 0.234) exhibited positive correlations with iEAT (see Supplemental File S1). Concerning HDL characteristics, iEAT showed a positive correlation with the content of apoC-III and a negative correlation with the content of apoAI (Table 3). No correlation was observed between iEAT and the function parameters of HDL.

Regarding LDL, the only significant correlation with iEAT was a negative one with LDL size (Table 4). Taken together, these correlations suggest a positive relationship between EAT accumulation and small LDL particles with increased atherogenic potential.

**Figure 9:**
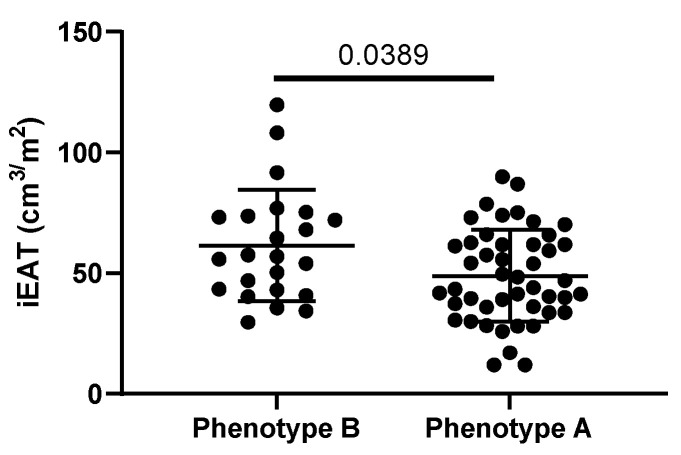
Indexed EAT (iEAT) volume in subjects showing phenotype B (LDL size < 26.0 nm) or phenotype A (LDL size > 26.0 nm) of the LDL subfraction pattern. iEAT was determined by MDCT analysis, and LDL size was assessed by nondenaturing polyacrylamide gradient gel electrophoresis, as described in Methods. The horizontal bar indicates a statistically significant difference between the groups. Data are mean ± SD.

To obtain further evidence of the association between LDL size and EAT, we compared the iEAT volume in subjects with phenotype B (prevalence of small LDL) with that of subjects with phenotype A (prevalence of large LDL particles) (Figure 9), presenting the former increased iEAT volume.

### 3.8. Bivariate and Multivariable Regression Analysis

To determine which variables could determine iEAT independently, bivariate linear regression analysis was performed using parameters that showed statistically significant correlations with iEAT. Among all the candidate biomarkers analyzed, age (*p* < 0.001), GGT (*p* < 0.001), LDL size (*p* = 0.002), BMI (*p* = 0.009), and HbA_1c_ (*p* = 0.049) were significantly associated with epicardial adipose tissue volume, as shown in Table 5. All the variables were positively associated except LDL size, whose association was negative.

Subsequently, a step-forward multivariable analysis, adjusted for hypoglucemic therapy, was conducted. Starting with age, BMI, and sex as classical variables, the forward stepwise multivariate sequentially added all the variables that showed associations with iEAT in the bivariate analysis (see Table 5). This analysis showed that age, LDL size, and GGT independently predicted iEAT accumulation (Table 6). These associations remained significant after adjusting for BMI and sex. Age, LDL size, and GGT explain approximately 50% of the variability in EAT volume. The resulting regression function obtained from this analysis was as follows:

iEAT = 84.83 + 1.18 × (age) + 0.425 × (GGT) − 4.15 × (LDL size)

### 3.9. Receiver Operating Characteristic Analysis

**Figure 10:**
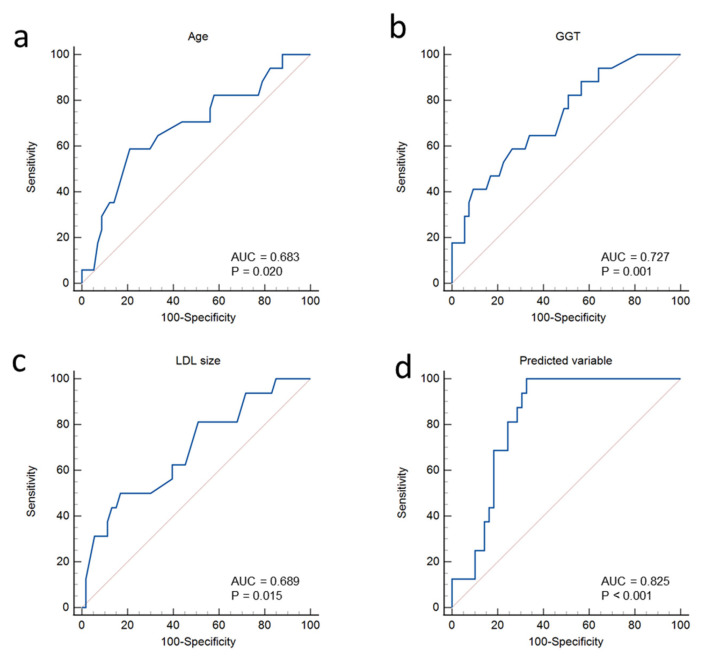
Receiver operating characteristic (ROC) analysis of (**a**) age, (**b**) GGT, and (**c**) LDL size for discrimination of iEAT ≥ 68.1 cm^3^/m^2^, considering these parameters independently. (**d**) ROC analysis, including age, LDL size, and GGT according to the equation: [iEAT = 84.83 + 1.18 × (age) + 0.425 × (GGT) − 4.15 × (LDL size)] for discrimination of iEAT ≥ 68.1 cm^3^/m^2^.

Shmilovich et al. placed the threshold value of iEAT for the prediction of major cardiovascular events at the 95th percentile (68.1 cm^3^/m^2^ of body surface area) in a population of healthy individuals [17]. ROC analysis was used to determine the sensitivity and specificity of different cut-off points of independently associated biomarkers for the discrimination of iEAT of 68.1 cm^3^/m^2^ or greater. The areas under the curve (AUCs) when age, LDL size, and GGT were considered independently were 0.683 (*p* = 0.023), 0.689 (*p* = 0.022), and 0.727 (*p* = 0.005), respectively, for the discrimination of patients with iEAT of 68.1 cm^3^/m^2^ or greater (Figure 10a–c).

The AUC of LDL size and GGT was below 0.8, suggesting a suboptimal predictive value when these biomarkers are used individually. Hence, we conducted another ROC analysis, which included age, LDL size, and GGT. In this case, we obtained an AUC of 0.835, which clearly improved the predictive value for the discrimination of patients with iEAT of 68.1 cm^3^/m^2^ or greater (Figure 10d). Details of this analysis are shown in Supplemental File S2. We observed that a cut-off point of 53.55 using the equation obtained in the multivariate analysis (iEAT = 84.83 + 1.18 × [age] + 0.425 × [GGT] − 4.15 × [LDL size]) predicted the risk of iEAT of 68.1 cm^3^/m^2^ or greater with a sensitivity of 100% and a specificity of 67.35%.

## 4. Discussion

The incidence of cardiovascular events, either due to atherosclerotic disease or derived from HF, is especially relevant in patients with diabetes and is closely related to abnormalities in lipid metabolism [30]. These patients, like others with metabolic disorders, such as obese individuals or those with metabolic syndrome, are characterized by the coexistence of diabetic dyslipidemia and a larger volume of EAT [31]. The accumulation of EAT underlies the high incidence of HF in diabetes and is related to cardiovascular disease and mortality in these patients [32]. However, despite the predictable interconnection that there must be between the presence of abnormalities in the lipoprotein profile and increases in EAT volume, few studies have addressed the impact of glycemic optimization in subjects with T2DM on EAT, and most have been conducted in nondiabetic subjects. Notably, the relationship, if any, between lipoprotein variables and EAT is limited to the quantitative characteristics obtained from conventional lipoprotein profiles [33,34,35,36]. In this context, our group previously reported an association between HDL composition and EAT volume in type 1 diabetic patients [37]. However, the qualitative properties of lipoproteins, including their composition and functionality, have not yet been studied in detail in T2DM patients.

Of note, based on clinical data and LVEF, NT-proBNP, TnT, and coronary calcium score values, the patients included in our study did not show evidence of advanced HF, coronary atherosclerosis, or renal disease. Regarding hepatic function, the levels of these parameters fell within what is considered normal liver function. In this context, our diabetic patients have not yet developed serious alterations that increase liver, kidney, heart, or vascular diseases. Nevertheless, the volume of accumulated EAT, which decreased after treatment, was clearly altered in the T2DM patients compared to the control group.

An apparently paradoxical observation was that CAC not only did not decrease, but showed a slight increase after improvement in glycemic control. This may be due to several reasons. The first is that CAC was only increased in five of the patients with diabetes, which implies a very low number of individuals. The other reason is that the time of 3–6 months of treatment could be too short to observe a regression of the atherosclerotic lesion. In fact, although there are certain discrepancies, generally the time to detect a decrease in the size of the lesion is several years after aggressive lipid-lowering treatment [38,39]. On the other hand, plaque regression does not necessarily imply a decrement of CAC score. It has been reported that in response to statins, fibrous and calcified plaque volumes appear to increase, whereas noncalcified, fibrofatty, and necrotic core volumes decrease [40]. This also could explain the results obtained in our study.

Our results show important quantitative and qualitative differences in lipoproteins between patients with diabetes and control individuals, with total or partial normalization of these alterations after the optimization of glycemic control. Quantitative alterations are well known and are characteristic of diabetic dyslipidemia, such as hypertriglyceridemia, hyper-apoB, and low levels of HDL [3]. Lp(a) is a parameter strongly related with coronary artery disease [41], but its relationship with EAT volume has been scarcely studied. In accordance with previous studies [42], no association between EAT and Lp(a) concentration was observed.

Regarding qualitative alterations, our data indicate larger VLDL particles, smaller LDL particles, and HDL with less apoA-I and more apoC-III content in T2DM samples with poor glycemic control, with most of these alterations amended after glycemic optimization.

The results presented here show that EAT volume is related not only to elevated levels of triglycerides and VLDLc but also to some abnormalities in the composition of HDL, and especially in the size of LDL. The association of hypertriglyceridemia and small dense LDL particles (sdLDL) with visceral adipose tissue has previously been demonstrated [43,44]; however, our study is the first to show a link between the increased accumulation of EAT and the predominance of sdLDL, a hallmark of diabetic dyslipidemia. The molecular mechanisms underlying this relationship remain unknown. A direct effect of sdLDL on the increase of EAT volume is unlikely, and it is most likely that the poor management of NEFA at the hepatic level, with the consequent formation of VLDL particles overloaded with triglycerides characteristic of hypertriglyceridemia, could favor the uptake of NEFA coming from VLDL by EAT. This could occur mainly for two reasons: (1) because triglyceride-enriched VLDL concentration is increased in diabetic plasma, and (2) because LpL activity in EAT is also increased in T2DM [45]. This would favor the uptake of fatty acids by EAT adipocytes, thereby increasing the volume of EAT. Alternatively, other putative harmful effects of sdLDL on EAT cannot be ignored. Thus, given the high susceptibility of sdLDL to modification [46], active lipid species could be generated, increasing the oxidative and inflammatory stress of EAT in subjects with atherogenic dyslipidemia, and perhaps favoring its growth. This could be related to the increased proportion of modified LDL(-) in T2DM patients with poor glycemic control observed in the current and previous studies [47]. This could be aggravated by the lower antioxidant capacity of HDL in diabetic patients with poor glycemic control, which could be related to the decreased proportion of PAF-AH in these particles. The putative effects of the abnormalities of the lipoprotein function and composition on the EAT function and volume should be addressed in future studies.

Another relevant aspect of our study was the relationship observed between sdLDL and plasma GGT concentration, as shown by the correlation analysis (*p* = 0.002, r: −0.370). This association has been described previously, although it was not in the context of diabetes [48]. The rationale for such a relationship is that increased GGT level is a marker of liver dysfunction, which would promote increased hepatic synthesis of triglyceride-enriched VLDL, ultimately leading to the formation of small, dense LDL particles in blood. In this context, the prevalence of sdLDL particles has been reported in patients with nonalcoholic fatty liver disease and nonalcoholic steatohepatitis [49,50]. On the other hand, GGT is recognized as a cardiovascular risk factor, and its plasma concentration has been associated with visceral adipose tissue and EAT in patients with coronary disease [51,52].

**Figure 11:**
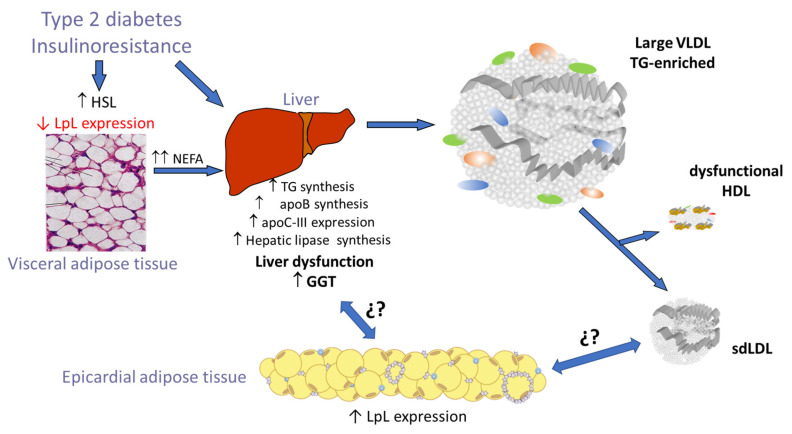
Putative mechanisms relating to sdLDL, GGT, and EAT volume in T2DM patients. In a situation of insulin resistance and increased visceral adiposity, there is a greater release of NEFA that is taken up by the liver. This would favor the increase in the production of VLDL loaded with triglycerides, the metabolic consequence of which is the formation of sdLDL particles and dysfunctional HDL. At the same time, there would be an increase in liver fat, which is reflected by an increase in the plasma levels of GGT. How the simultaneous existence of hepatic dysfunction and the presence of sdLDL could favor the increase in EAT volume requires more detailed studies to elucidate the molecular mechanisms involved. ¿?, unknown metabolic pathways; ↑ increase; ↓ decrease.

Taken together, these findings point to a close interrelationship between sdLDL, GGT, and EAT volume, supporting the hepatic–myocardial axis as a key player in the development of heart failure and coronary atherosclerosis. Figure 11 summarizes the main findings of this study. In a situation of insulin resistance, such as in T2DM, visceral adipose tissue releases high quantities of NEFA that are taken up by the liver. This would favor hepatic lipid accumulation and the formation of large VLDL particles enriched in triglycerides, which, after blood catabolism, generates dysfunctional HDL particles and sdLDL. Hence, increased sdLDL and GGT levels reflect a situation in which the enlargement of EAT is triggered, which could result in the early development of heart failure and coronary atherosclerosis. The molecular mechanisms by which sdLDL and liver dysfunction promote EAT growth are still unknown and warrant further study.

Together with age, LDL size and GGT are the main predictors of increased EAT volume. These three variables explain approximately 50% of the variability in EAT volume. This opens up the possibility of using an equation that includes these variables to predict the volume of EAT, and therefore the risk of developing HF in the future. In this way, the use of expensive and complex image analysis could be reduced as much as possible.

Our study has several limitations. The first is the small number of patients analyzed, which, in our case, was due to the fact that we did not limit ourselves to quantifying the size of LDL and GGT in plasma; rather, we wanted to perform an exhaustive analysis of other biological properties of lipoproteins. Thus, although the present study suggests that LDL size, GGT and age can be used to estimate EAT volume, these results should be taken with caution, and only considered valid if corroborated by other studies. Therefore, larger studies are necessary to validate the usefulness of LDL size and GGT level measurement as predictors of EAT volume in diabetes. In addition, our data must also be validated in other groups of patients with other pathologies to determine if the results can be extrapolated to the general population. The second limitation is that in our study, we used nondenaturing acrylamide gradient electrophoresis to measure the size of LDL, a method that is complex and not available to most health-care laboratories. Therefore, it is necessary to confirm our data using other, simpler methods for measuring LDL size. The appearance on the market of automatable methods to quantify sdLDL concentration is a very reasonable, simple, and cheap possibility [53,54]. Alternatively, the concentration of sdLDL can be determined by MRI [55,56], which is a very resolute method but has the disadvantages of high cost and availability only in highly specialized laboratories.

On the other hand, a strength of our study that we want to highlight is that it was carried out in patients with type 2 diabetes without evidence of advanced heart failure, coronary stenosis or hepatic disease. This suggests that the interrelation that we observed among sdLDL concentration, GGT level, and EAT volume occurs at a stage prior to the development of these pathologies, so they would be very useful in their prevention.

## 5. Conclusions

In summary, our data suggest that LDL size, as a representative of atherogenic dyslipemia, and GGT, as a marker of liver dysfunction, are closely related to increased EAT volume and could be independent predictors of cardiovascular risk. Further studies are necessary to confirm this relationship and to validate the possibility of using these parameters to predict increased EAT volume and the risk of CAD and heart failure.

## Figures and Tables

**Table 1:** Clinical and anthropometric characteristics, and hypoglycemic treatment of patients with diabetes and control subjects.

	HC (n = 14)	PGC (n = 36)	GGC (n = 36)
Age (years)	53.8 ± 5.23	55.8 ± 9.41	
Sex (m/f)	10/4 (71.4)	25/11	
BMI (kg/m^2^)	28.28 ± 4.98	33.53 ± 7.27 ^#^	31.87 ± 5.59 *^#^
Weight (kg)	81.72 ± 15.55	95.07 ± 19.38 ^#^	90.05 ± 14.18 *^#^
WC (cm)	97.14 ± 8.84	109.41 ± 14.68 ^#^	107.59 ± 11.61 *^#^
Smoking habit	4 (28.6)	10 (27.8)	10 (27.8)
Dyslipidemia	0 (0)	12 (33.3) ^#^	10 (27.8)
Hypertension	2 (14.3)	12 (33.3) ^#^	12 (33.3)
Hypoglycemic treatment			
Metformin	-	-	30
Empaglifozin	-	-	33
aGLP1	-	-	2
DPP4i	-	-	4

HC: healthy controls; PGC: poor glycemic control; GGC: good glycemic control. aGLP1: glucagon-like peptide 1 (GLP1-rA) agonist; DPP4i: Dipeptidyl peptidase 4 (DPP-4) inhibitor; WC: waist circumference. Data are expressed as mean ± SD or n (%). * *p* < 0.05 vs. PGC and ^#^ *p* < 0.05 vs. HC.

**Table 2:** Biochemical and lipid profiles of patients with diabetes and control subjects.

	HC (n = 14)	PGC (n = 36)	GGC (n = 36)
HbA1c (%)	5.4 ± 0.2	11.7 ± 2.1 ^#^	6.1 (0.77) *^#^
Glucose (mg/dL)	88.9 ± 10.2	157.5 ± 66.6 ^#^	117.2 ± 20.3 *^#^
C-peptide (pmol/L)	nd	841 (642.1)	1034 (301.5)
Bilirubin (µmol/L)	12.5 ± 4.8	10.8 ± 4.3	10.5 ± 3.6
AST (U/L)	22.62 ± 4.3	34.4 ± 17.7 ^#^	22.9 ± 13.2 *^#^
ALT (U/L)	22.64 ± 9.1	42.9 ± 22.8 ^#^	27.9 ± 19.0 *^#^
ALP (U/L)	70.4 ± 12.8	97.5 ± 25.5 ^#^	84.9 ± 19.1 *^#^
GGT (U/L)	24.9 ± 11.4	52.6 ± 62.2 ^#^	35.2 ± 41.6 *^#^
CRP (mg/L)	1.3 (1.72)	7.7 (7.4) ^#^	2.35 (5.05) *^#^
**Lipid profile**			
Cholesterol (mg/dL)	194.6 ± 39.84	189.84 ± 37.61	180.91 ± 47.92
Triglyceride (mg/dL)	69.48 (61.06)	141.6 (101.82) ^#^	132.8 (67.26) ^#^
HDLc (mg/dL)	54.46 ± 12.31	40.08 ± 9.37 ^#^	45.03 ± 8.47 *^#^
LDLc (mg/dL)	123.01 ± 33.04	119.80 ± 31.65	105.56 ± 37.82
VLDLc (mg/dL)	14.0 (12.3)	31.01 ± 12.11 ^#^	26.77 (13.02) ^#^
Lp(a) (mg/L)	241.7 ± 215.1	234.5 ± 277.3	266.9 ± 297.7
**Apolipoprotein**			
ApoB (g/L)	0.93 ± 0.25	1.07 ± 0.26 ^#^	0.94 ± 0.31
ApoA-I (g/L)	1.65 ± 0.26	1.31 ± 0.23 ^#^	1.43 ± 0.24 *^#^
ApoA-II (mg/L)	43.83 ± 4.93	34.19 ± 6.53 ^#^	37.42 ± 6.31 *^#^
ApoC-III (mg/L)	8.22 (6.58)	7.61 (6.64)	8.57 (8.8)
ApoE (mg/L)	5.76 ± 1.60	5.85 ± 1.81	5.33 ± 1.75

PGC: poor glycemic control; GGC: good glycemic control; HC: healthy controls. Data are expressed as mean ± SD, median ± IQR, or n (%). * *p* < 0.05 vs. PGC and ^#^ *p* < 0.05 vs. HC. nd, not determined.

**Table 3:** Spearman’s rank correlation coefficient test of iEAT with HDL-related parameters.

HDL Composition	*p*	r
% cholesterol	0.597	−0.063
% triglycerides	0.268	0.133
% phospholipids	0.252	0.138
% free cholesterol	0.072	−0.214
% esterified cholesterol	0.847	0.023
% apo A-I	**0.023**	**−0.268**
% apo A-II	0.707	0.045
% apo E	0.391	−0.103
% apo C-III	**0.035**	**0.251**
**HDL Function**		
Total PAF-AH	0.356	0.111
PAF-AH in HDL (nmol/min/mL)	0.176	0.162
PAF-AH in HDL (%)	0.25	0.137
HDL2	0.901	0.015
HDL3	0.901	−0.015
Antioxidant capacity of HDL	0.584	−0.067
HDL susceptibility to oxidation	0.738	−0.041

Statistically significant correlations are indicated in bold type.

**Table 4:** Spearman’s rank correlation coefficient test of iEAT with LDL-related parameters.

LDL Composition	*p*	r
% cholesterol	0.848	−0.023
% triglycerides	0.822	−0.027
% phospholipids	0.495	0.082
% free cholesterol	0.068	−0.217
% esterified cholesterol	0.360	0.110
% apo A-I	0.789	−0.032
% apo E	0.501	−0.081
% apo C-III	0.755	−0.037
**LDL Function**		
LDL susceptibility to aggregation	0.258	−0.135
LDL(-)	**0.807**	**−0.029**
LDL size	**0.007**	**−0.320**
LDL susceptibility to oxidation	**0.252**	**−0.143**

Statistically significant correlations are indicated in bold type.

**Table 5:** Bivariate linear regression analysis with iEAT.

Bivariate Analysis	β	*p*
Age	0.413	**<0.001**
BMI	0.300	**0.009**
GGT	0.467	**<0.001**
LDLsize	−0.372	**0.002**
HbA1c	0.237	**0.049**
Tg	0.189	0.111
%ApoAI in HDL	−0.22	0.065
%ApoCIII in HDL	0.198	0.098

Statistically significant associations with iEAT are indicated in bold type.

**Table 6:** Step-forward multivariable linear regression analysis.

	β	*p*	R^2^
Age	0.484	**<0.001**	0.505
Sex	0.164	0.085	
BMI	0.164	0.089	
LDL size	−0.268	**0.01**	
GGT	0.343	**0.001**	

Statistically significant associations with iEAT are indicated in bold type.

## Data Availability

All the information of this study is available upon reasonable request by contacting with the corresponding author.

## Supplementary Materials

The following supporting information can be downloaded at: https://www.mdpi.com/article/10.3390/jcm14030862/s1, Supplemental File S1: Correlations; Supplemental File S2: ROC analysis.
