# Supplementary material for: Low-Density Lipoprotein Subfraction Phenotype Is Associated with Epicardial Adipose Tissue Volume in Type 2 Diabetes
Source: J Clin Med. 2025 Jan 28;14(3):862. doi: 10.3390/jcm14030862 (PMC11818426; doi:10.3390/jcm14030862)
Supplement: Supplementary file 1 [file jcm-14-00862-s001.zip › Supplementary file 2 ROC predicted variable.pdf]

ROC curve

Variable	Predicted_variable<br>Predicted variable
Classification variable	iEAT

Sample size	65
Positive group [a]	16 (24,62%)
Negative group [b]	49 (75,38%)

[a] iEAT = 1
[b] iEAT = 0

Disease prevalence (%)	unknown

Area under the ROC curve (AUC)

Area under the ROC curve (AUC)	0,825
Standard Error [a]	0,0500
95% Confidence interval [b]	0,711 to 0,908
z statistic	6,502
Significance level P (Area=0.5)	<0,0001

[a] DeLong et al., 1988
[b] Binomial exact

Youden index

Youden index J	0,6735
Associated criterion	>53,55
Sensitivity	100,00
Specificity	67,35

Criterion values and coordinates of the ROC curve [Hide]

Criterion	Sensitivity	95% CI	Specificity	95% CI	+LR	-LR
≥17,89	100,00	79,4 - 100,0	0,00	0,0 - 7,3	1,00	
>17,89	100,00	79,4 - 100,0	2,04	0,05 - 10,9	1,02	0,00
>24,05	100,00	79,4 - 100,0	4,08	0,5 - 14,0	1,04	0,00
>29,91	100,00	79,4 - 100,0	6,12	1,3 - 16,9	1,07	0,00
>30,17	100,00	79,4 - 100,0	8,16	2,3 - 19,6	1,09	0,00
>31,1	100,00	79,4 - 100,0	10,20	3,4 - 22,2	1,11	0,00
>31,22	100,00	79,4 - 100,0	12,24	4,6 - 24,8	1,14	0,00
>31,59	100,00	79,4 - 100,0	14,29	5,9 - 27,2	1,17	0,00
>35,17	100,00	79,4 - 100,0	16,33	7,3 - 29,7	1,20	0,00
>36,94	100,00	79,4 - 100,0	18,37	8,8 - 32,0	1,22	0,00
>38,49	100,00	79,4 - 100,0	20,41	10,2 - 34,3	1,26	0,00
>38,6	100,00	79,4 - 100,0	22,45	11,8 - 36,6	1,29	0,00
>38,61	100,00	79,4 - 100,0	24,49	13,3 - 38,9	1,32	0,00
>39,09	100,00	79,4 - 100,0	26,53	14,9 - 41,1	1,36	0,00
>39,17	100,00	79,4 - 100,0	28,57	16,6 - 43,3	1,40	0,00
>39,74	100,00	79,4 - 100,0	30,61	18,3 - 45,4	1,44	0,00
>39,75	100,00	79,4 - 100,0	32,65	19,9 - 47,5	1,48	0,00
>40,25	100,00	79,4 - 100,0	34,69	21,7 - 49,6	1,53	0,00

>40,75	100,00	79,4 - 100,0	36,73	23,4 - 51,7	1,58	0,00
>40,93	100,00	79,4 - 100,0	38,78	25,2 - 53,8	1,63	0,00
>42,53	100,00	79,4 - 100,0	40,82	27,0 - 55,8	1,69	0,00
>43,37	100,00	79,4 - 100,0	42,86	28,8 - 57,8	1,75	0,00
>46,65	100,00	79,4 - 100,0	44,90	30,7 - 59,8	1,81	0,00
>46,71	100,00	79,4 - 100,0	46,94	32,5 - 61,7	1,88	0,00
>46,73	100,00	79,4 - 100,0	48,98	34,4 - 63,7	1,96	0,00
>46,92	100,00	79,4 - 100,0	51,02	36,3 - 65,6	2,04	0,00
>48,33	100,00	79,4 - 100,0	53,06	38,3 - 67,5	2,13	0,00
>48,85	100,00	79,4 - 100,0	55,10	40,2 - 69,3	2,23	0,00
>48,96	100,00	79,4 - 100,0	57,14	42,2 - 71,2	2,33	0,00
>49,98	100,00	79,4 - 100,0	59,18	44,2 - 73,0	2,45	0,00
>50,69	100,00	79,4 - 100,0	61,22	46,2 - 74,8	2,58	0,00
>50,86	100,00	79,4 - 100,0	63,27	48,3 - 76,6	2,72	0,00
>50,87	100,00	79,4 - 100,0	65,31	50,4 - 78,3	2,88	0,00
>53,55	100,00	79,4 - 100,0	67,35	52,5 - 80,1	3,06	0,00
>54,01	93,75	69,8 - 99,8	67,35	52,5 - 80,1	2,87	0,093
>55,08	93,75	69,8 - 99,8	69,39	54,6 - 81,7	3,06	0,090
>55,31	87,50	61,7 - 98,4	69,39	54,6 - 81,7	2,86	0,18
>56,06	87,50	61,7 - 98,4	71,43	56,7 - 83,4	3,06	0,18
>56,21	81,25	54,4 - 96,0	71,43	56,7 - 83,4	2,84	0,26
>57,86	81,25	54,4 - 96,0	73,47	58,9 - 85,1	3,06	0,26
>57,94	81,25	54,4 - 96,0	75,51	61,1 - 86,7	3,32	0,25
>58,13	75,00	47,6 - 92,7	75,51	61,1 - 86,7	3,06	0,33
>58,24	68,75	41,3 - 89,0	75,51	61,1 - 86,7	2,81	0,41
>58,6	68,75	41,3 - 89,0	77,55	63,4 - 88,2	3,06	0,40
>58,92	68,75	41,3 - 89,0	79,59	65,7 - 89,8	3,37	0,39
>59,55	68,75	41,3 - 89,0	81,63	68,0 - 91,2	3,74	0,38
>62,38	62,50	35,4 - 84,8	81,63	68,0 - 91,2	3,40	0,46
>63,38	56,25	29,9 - 80,2	81,63	68,0 - 91,2	3,06	0,54
>64,26	50,00	24,7 - 75,3	81,63	68,0 - 91,2	2,72	0,61
>65,42	43,75	19,8 - 70,1	81,63	68,0 - 91,2	2,38	0,69
>65,72	43,75	19,8 - 70,1	83,67	70,3 - 92,7	2,68	0,67
>65,99	37,50	15,2 - 64,6	83,67	70,3 - 92,7	2,30	0,75
>66,06	37,50	15,2 - 64,6	85,71	72,8 - 94,1	2,62	0,73
>66,22	31,25	11,0 - 58,7	85,71	72,8 - 94,1	2,19	0,80
>67,24	25,00	7,3 - 52,4	85,71	72,8 - 94,1	1,75	0,88
>67,42	25,00	7,3 - 52,4	87,76	75,2 - 95,4	2,04	0,85
>71,3	25,00	7,3 - 52,4	89,80	77,8 - 96,6	2,45	0,84
>71,83	18,75	4,0 - 45,6	89,80	77,8 - 96,6	1,84	0,90
>72,44	12,50	1,6 - 38,3	89,80	77,8 - 96,6	1,22	0,97
>73,73	12,50	1,6 - 38,3	91,84	80,4 - 97,7	1,53	0,95
>73,74	12,50	1,6 - 38,3	93,88	83,1 - 98,7	2,04	0,93
>73,9	12,50	1,6 - 38,3	95,92	86,0 - 99,5	3,06	0,91
>74,22	12,50	1,6 - 38,3	97,96	89,1 - 99,9	6,12	0,89
>75,84	12,50	1,6 - 38,3	100,00	92,7 - 100,0		0,88
>84,93	6,25	0,2 - 30,2	100,00	92,7 - 100,0		0,94
>97,66	0,00	0,0 - 20,6	100,00	92,7 - 100,0		1,00

C:\Users\jrive\OneDrive\Escritorio\medcalc file.mc1

Monday, March 25, 2024 18:04

MedCalc® Statistical Software version 22.021 (MedCalc Software Ltd, Ostend, Belgium; https://www.medcalc.org; 2024)
